# Alcohol Exposure Impacts the Composition of HeLa-Derived Extracellular Vesicles

**DOI:** 10.3390/biomedicines7040078

**Published:** 2019-09-30

**Authors:** Leandra B. Jones, Sanjay Kumar, Aliyah J. Curry, Jayde S. Price, Alexandre Krendelchtchikov, Brennetta J. Crenshaw, Courtnee’ R. Bell, Sparkle D. Williams, Tambre A. Tolliver, Sabita N. Saldanha, Brian Sims, Qiana L. Matthews

**Affiliations:** 1 Microbiology Program, Department of Biological Sciences, College of Science, Technology, Engineering and Mathematics, Alabama State University, Montgomery, AL 36104, USA; ljones@alasu.edu (L.B.J.); bjcrenshaw0320@gmail.com (B.J.C.); courtneerbell@yahoo.com (C.R.B.); 2 Departments of Pediatrics and Cell, Developmental and Integrative Biology, Division of Neonatology, University of Alabama at Birmingham, Birmingham, AL 35294, USA; skumar@peds.uab.edu (S.K.); alex.krend@gmail.com (A.K.); swilliams@vt.edu (S.D.W.); bsims@peds.uab.edu (B.S.); 3 Department of Biological Sciences, College of Science, Technology, Engineering and Mathematics, Alabama State University, Montgomery, AL 36104, USA; aliyahcurry6@gmail.com (A.J.C.); JPRICE01@auburn.vcom.edu (J.S.P.); tamtoll93@gmail.com (T.A.T.); ssaldanha@alasu.edu (S.N.S.); 4 Center for Nanobiotechnology Research (CNBR), Alabama State University, Montgomery, AL 36104, USA

**Keywords:** HeLa cells, cervical cancer, alcohol, extracellular vesicles, exosomes, tetraspanins

## Abstract

Extracellular vesicles are nanosized vesicles that are under intense investigation for their role in intercellular communication. Extracellular vesicles have begun to be examined for their role in disease protection and their role as disease biomarkers and/or vaccine agents. The goal of this study was to investigate the effects of alcohol exposure on the biogenesis and composition of extracellular vesicles derived from the cervical cancer line, HeLa. The HeLa cells were cultured in exosome-free media and were either mock-treated (control) or treated with 50 mM or 100 mM of alcohol for 24 h and 48 h. Our results demonstrated that alcohol significantly impacts HeLa cell viability and exosome biogenesis/composition. Importantly, our studies demonstrate the critical role of alcohol on HeLa cells, as well as HeLa-derived extracellular vesicle biogenesis and composition. Specifically, these results indicate that alcohol alters extracellular vesicles’ packaging of heat shock proteins and apoptotic proteins. Extracellular vesicles serve as communicators for HeLa cells, as well as biomarkers for the initiation and progression of disease.

## 1. Introduction

The World Health Organization (WHO)-International Agency for Research on Cancer (IARC) (2009) has classified alcoholic beverages as a Group 1 Carcinogen (carcinogenic to humans). A study completed by Madsen et al. found that in the absence of alcohol consumption, the prevalence of vaginal squamous cell carcinomas (VV-SCC) greatly decreased [1]. Madsen et al. also found a higher risk of VV-SCC in women that had high alcohol consumption [1]. With the high mortality rate of cervical cancers, more information is needed regarding detection and treatment of the diseased. In this regard, several investigators have begun to examine extracellular vesicles (EVs) and their role in disease protection [2], as disease biomarkers [3,4,5] and/or as a cargo/vaccine agents for cervical cancer [6,7,8,9,10,11,12,13,14,15,16,17,18].

Recent studies demonstrated that body fluids contain substantial amounts of EVs with sizes varying between 30 and 1000 nm. Three main types of EVs have been described. First, nanosized exosomes (30–150 nm), which are derivatives of the endosomal system; second, microvesicles (100–1000 nm), which are produced from outward budding of the plasma membrane [19]; third, apoptotic bodies (ABs) are the largest extracellular vesicles. ABs, whose size varies between 1 and 5 µm, are released by apoptotic cells as blebs [20,21,22]. AB secretion serves as a signal stimulating phagocytosis of apoptotic cells before the induction of secondary necrosis [21,23]. Due to the small differences in physical properties and composition, discrimination between extracellular vesicle (EV) populations remains difficult. Further complicating the distinction of the types of vesicles is the fact that the same type of cell might secrete multiple types of EVs [19].

Discovered in the early 1980s, nanosized exosomes are heterogeneously-shaped vesicles that are released from the cell’s plasma membrane via exocytosis into extracellular environments [24,25]. Exosomes can facilitate intercellular communication, by carrying out endocytosis of vesicles, and horizontally transferring secreted molecules such as DNA [26], RNA (miRNA, mRNA) [27], and proteins [28]. These nanosized vesicles are released by healthy cells, cells undergoing apoptosis, and cells under stress (i.e., hypoxia, drugs, alcohol, bacterial, and viral infections) [25,29,30,31]. Exosomes have been found in the plasma, urine, cerebrospinal fluid, saliva, breast milk, amniotic, and bronchoalveolar lavage fluid [25,32]. They can be secreted by a large variety of cells, such as mast cells, dendritic cells, T cells, B cells, stem cells, astrocytes, endothelial cells, tumor cells, and epithelial cells [24,25,32]. Exosomes have several different cell surface molecules and are able to activate many cell receptors, which allow them to participate in the exchange of materials between cells (i.e., proteins, lipids, carbohydrates, and pathogens) [26]. Exosomes can be used as biomarkers or certain vaccine vehicles for diseases [3,4,5]. The ability of exosomes to transfer information to uninfected cells for intercellular communication makes them an ideal candidate to treat diseases by delivering a desired agent or substance.

Exosomes are released by most cell types and mediate targeting in normal cells under physiological and pathophysiological conditions, including different types of cancer [19,33,34]. Elevated levels of EV-expressing TYRP-2, VLA-4, and heat shock proteins (Hsp) 70 and 90 have been detected in the plasma of melanoma patients [35]. Hsp70 and Hsp90 have been investigated in order to understand their potential role in cancer pathogenesis and progression. Of important note, EV-associated levels of Hsp60 were dramatically decreased in colon cancer patients after surgically removing the tumor [36]. This suggests the importance of circulating EVs quantification as the follow-up of surgical treatment of cancers, both at the diagnosis and following relapse. The majority of the available clinical data supporting the use of EVs including exosomes, as a source of disease biomarkers, have been obtained from studies in a cohort of cancer patients [19]. These data are of paramount importance in the management of tumor patients and serve an unmet need. These data are important for two reasons. First, knowing that cancer initiation and cancer progression is multifactorial, better diagnostic tools such as exosomes are needed [37,38]. Secondly, knowing that cancer progression can be further exasperated by other external stimuli (i.e., nicotine and alcohol) further reiterates the need for better diagnostic tools [35]. Cappello and colleagues organized an elegant review that detailed important data illustrating the utility of exosomes in clinical studies [39]; however, this review highlighted the limited in vitro and clinical data related to the utility of exosomes and gynecological cancers [40]. As a model system, here, we evaluated the affect of alcohol administration over time on the cervical cancer cell line, HeLa. In addition, we observed the effect of alcohol administration on HeLa-derived EV biogenesis and composition.

## 2. Materials and Methods

### 2.1. Cell Culture

Human cervical epithelial (HeLa) cells were purchased from American Type Culture Collection (ATCC) (Manassas, Virginia, USA) and cultured in Dulbecco’s Modified Eagle Medium: Nutrient Mixture F-12 (DMEM/F12) with 10% fetal bovine serum (FBS), 10 mM L-glutamine, and 1% penicillin-streptomycin (all from Life Technologies, Grand Island, NY, USA). Exosome-free DMEM/F12 media was made in the same manner as DMEM/F12 using exosome-free FBS from System Biosciences (Palo Alto, CA, USA). The cell line was incubated at 37 °C and 5% CO_2_ under standard humidified conditions.

### 2.2. Cell Ethanol Dosing

HeLa cells were washed with 5 mL of sterile 1× phosphate-buffered saline (PBS) (Fisher Scientific, Hampton, NH, USA), removed with 0.05% trypsin) (Fisher Scientific, Hampton, NH, USA), and plated at 5 × 10^5^ cells/per dish with 6 mL of DMEM/F12) (Fisher Scientific, Hampton, NH, USA). The following day, media was removed and replaced with exosome-free DMEM/F12. The dishes were dosed with DMEM/F12 exosome-free media only (control treatment) and DMEM/F12 exosome-free media containing 200-proof 50 mM and 100 mM EtOH (Millipore Sigma, St. Louis, MO, USA). The media was collected at 24 h or 48 h.

### 2.3. Cell Viability by Trypan Blue Exclusion

HeLa cells were grown to 70–80% confluency and seeded into tissue culture dishes at 5 × 10^5^ cells/per dish with 6 mL of DMEM/F12. The following day, media was removed, and 6 mL exosome-free DMEM/F12 were added to the dishes. Cells were treated with 0.05% trypsin, scraped, and collected at 24 h or 48 h. Cells were stained with 0.4% trypan blue solution, and viability was measured using the trypan blue dye in Cell Countess (Fisher Scientific, Hampton, NH, USA). Percent viability = [1.00 − (number of blue cells ÷ number of total cells)] × 100.

### 2.4. EV Purification and Isolation

EVs were isolated from the media following EtOH exposure at different concentrations. EVs were also isolated from cells not treated and used as controls. Four to five milliliters of conditioned medium was collected for exosome purification. In brief, extracellular vesicles were isolated as previously described [31]. After collecting media, the media was spun down at 300× *g* at 4 °C for 10 min using a Sorvall^©^ RT 6000 refrigerated centrifuge (Sorvall, Ontario, Canada). The media was collected, and the cell pellet was discarded. The media was spun again at 2600× *g* at 4 °C for 10 min using a Sorvall© RT 6000 refrigerated centrifuge. The media was then filtered through a 10-mL syringe with a 25-mm syringe filter, with a porosity of 0.22 µm. Six milliliters of PBS was added to the media and centrifuged at 20,000× *g* for 45 min in a SW41T1 swinging bucket rotor at 4 °C using a Beckman Coulter Optima ™ L-70K Ultracentrifuge (Beckman Couter, IN, USA). To collect the EVs, the media was collected and centrifuged for 110,000× *g* for 70 min in a SW41T1 swinging bucket rotor at 4 °C using a Beckman Coulter Optima ™ L-70K Ultracentrifuge. The supernatant was removed, and approximately 500 µL of resuspended EVs were recovered from the sample. Isolated EVs were quantitated using Lowry quantitation methods [41].

### 2.5. EV Analysis by NTA

The size of HeLa-derived EVs was determined by Nanosight tracking analysis (NTA), by measuring Brownian motion to particle size distribution and count, using the Nanosight NS300 Sub-micron Particle Imaging System (Malvern, UK). Ten milliliters of 1× PBS diluted (1:1000) samples were injected into the machine sample chamber, and EVs were measured at room temperature (RT). The analysis of data was completed and recorded by the NTA software. The means ± SEM were recorded and analyzed for each given reading frame of the five independent experiments.

### 2.6. Sodium Dodecyl Sulfate–Polyacrylamide Gel Electrophoresis and Western Blot Analyses

To analyze EV-associated proteins, EVs were mixed with 5× sample loading buffer, boiled, and resolved on a 4–12% Bis-Tris sodium dodecyl sulfate gel, followed by transfer and blocking on a polyvinylidene difluoride membrane (Bio-Rad Laboratories, Hercules, CA, USA). Blotting was performed with clathrin and Hsp70 primary antibodies (Clathrin, 1:1000 dilution, BD Biosciences, East Rutherford, NJ, USA or Hsp70, 1:1000 dilution, Fisher Scientific, Waltham, MA, USA). Incubation with secondary antibody was performed using horseradish peroxidase (HRP)-conjugated goat anti-mouse 1:2,000 dilution (Millipore, Burlington, MA, USA). Proteins were detected using an enhanced chemiluminescence kit (ELC Western Blotting Substrate Pierce/Thermo Fisher Scientific Waltham, MA, USA) and the signals were developed on a Bio-Rad ChemiDoc™ XRS+ System (Bio-Rad Laboratories, Hercules, CA, USA).

### 2.7. Dot Blot Analysis

Cell lysates were evaluated via dot blot analysis. Zero-point-eight micrograms of cell lysate were lysed with lysis buffer (Lane Marker Reducing Sample Buffer, Fisher Scientific, Hampton, NH, USA), boiled, and bound to nitrocellulose membranes for 10 min. Samples were blocked in Pierce Fast-Blocker (Fisher Scientific, Hampton, NH, USA) with 0.09% Tween-20 for 5 min. After blocking, primary antibodies Rab 5 (1: 500 dilution, Thermo Fisher Scientific, Waltham, MA, USA) and Rab 7 (1:500 dilution, Thermo Fisher Scientific, Waltham, MA, USA USA) were added to the samples for incubation. Samples were incubated for 1 h at RT. Nitrocellulose blots were washed three times with 0.09% Tween-20 in 1× PBS for 10 min. Goat anti-rabbit (H+L) secondary antibody HRP (1:1000 dilution, Novus) was added in blocking solution (Pierce Fast-Blocker (Fisher Scientific, Hampton, NH, USA) with 0.09% Tween-20 in 1× PBS) for 1 h of shaking at RT. The blots were washed three times with 0.09% Tween-20 in 1× PBS for 10 min. The nitrocellulose membranes were developed using Super Signal West Femto Maximum Sensitivity Substrate (Thermo Fisher Scientific, Waltham, MA, USA). The signals were developed on a Bio-Rad ChemiDoc™ XRS+ System (Bio-Rad Laboratories, Hercules, CA, USA).

### 2.8. Enzyme-Linked Immunosorbent Assay

In order to investigate EV-associated proteins, ELISAs were performed. ELISA plates were coated with EVs collected from 24 h or 48 h with concentrations of 0 mM, 50 mM, and 100 mM or control (blocking buffer). Forty milligrams of samples were bound overnight at 4 °C on a 96-well plate with bicarbonate buffer (pH 9.5). EVs were blocked for 1 h in 0.05% BSA + Tween-20 (blocking buffer) at 4 °C. Incubated plates were then washed three times, and the detection antibody was prepared. One hundred microliters of primary antibody (CD81, 1:1000 dilution, Invitrogen, Waltham, MA, USA; Hsp60, 1:1000 dilution, Thermo Fisher Scientific, Waltham, MA, USA; Hsp70, 1:1000 dilution, Thermo Fisher Scientific, Waltham, MA, USA; Hsp90β 1:500 dilution, Thermo Fisher Scientific, Waltham, MA, USA; FAS, 1:1000 dilution, Thermo Fisher Scientific, Waltham, MA, USA; cleaved caspase 9, 1:1000 dilution, Cell Signaling, Danvers, MA, USA) with blocking buffer were then added and incubated at RT for 2 h. ELISA plates were then washed three times, and secondary antibody, HRP-conjugated goat anti-mouse (1:5000 dilution, Dako, MA, USA) or HRP-conjugated goat anti-rabbit (1:2000 dilution, Dako, MA, USA) was added. After 2 h of RT incubation, ELISAs were developed with SIGMAFAST™ *o*-phenylenediamine dihydrochloride (OPD) peroxidase substrate (Millipore Sigma, MO, USA) and measured at OD 405 nm using an automated ELISA plate reader.

### 2.9. Statistical Data

Descriptive statistics were calculated to study various variables of importance (i.e., means). Statistics were performed using Graph pad, version 5 (San Diego, CA, USA) one-way ANOVA or with a two-tailed distribution and two-sample unequal variance with post hoc Tukey’s analysis. Statistical significance was defined as follows: * *p* ≤ 0.05, ** *p* ≤ 0.01, and *** *p* ≤ 0.001.

## 3. Results

### 3.1. Cell Viability of HeLa Cells after EtOH Administration

**Figure 1:**
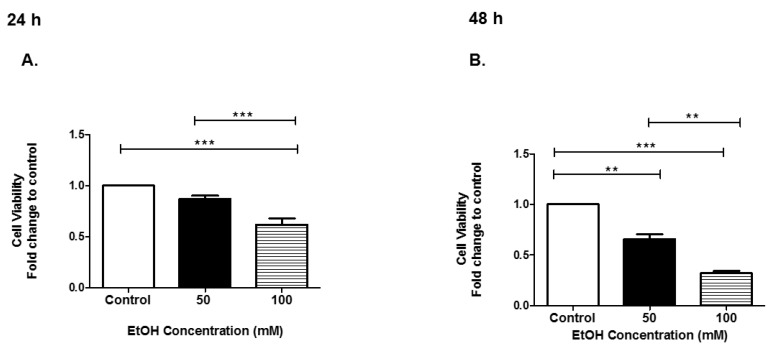
Effect of EtOH on HeLa cells. HeLa cells were treated with EtOH for (**A**) 24 h or (**B**) 48 h. At 24 h and 48 h, EtOH administration cell viability was assessed by the trypan blue exclusion assay. Data are presented as the means ± SEM of five independent experiments. Significance is defined as ** *p* ≤ 0.01, *** *p* ≤ 0.001.

To determine the effect of EtOH on the HeLa cell line, the following experiments were performed. HeLa cells not treated (control) or treated with EtOH at 50 mM or 100 mM in the exosome-free medium for 24 h and 48 h were evaluated for cell viability. The trypan blue exclusion assay was performed to determine the number of viable cells present at 24 h or 48 h of EtOH administration. At 24 h, HeLa cell viability significantly decreased when EtOH was administered (Figure 1A). At 48 h, HeLa cell viability significantly decreased when EtOH was administered (Figure 1B). At 48 h, 50 mM, cell viability was significantly decreased by 0.35-fold (*p* ≤ 0.01) and 0.68-fold (*p* ≤ 0.001) at 100 mM EtOH treatment, compared to control-treated cells Figure 1B. In summary, EtOH dramatically impacted HeLa cell viability at both time points.

### 3.2. Characterization of EVs

**Figure 2:**
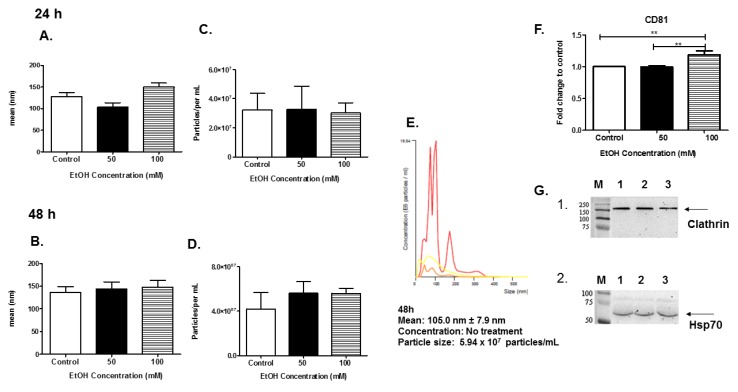
Validation of HeLa-derived EVs. Nanosight tracking analysis (NTA)-generated (**A**,**B**) size and (**C**,**D**) concentration of vesicles obtained from five independent experiments after EtOH administration. (**E**) Histogram plot of control EVs collected at 48 h. The different colored lines represent different pools of EVs. (**F**) CD81 was observed in HeLa-derived EVs via ELISA. (**G**) Western blots of HeLa-derived EVs, 60 μg/lane, were probed with (1) clathrin or (2) Hsp70 antibodies. Arrows indicate proteins of interest. Significance is defined as ** *p* ≤ 0.01.

EVs were isolated and characterized using NTA. The EVs released into the culture media were isolated and purified for biophysical analyses. EV particle size was determined using NTA (Figure 2A,B). In this system, the EVs were visualized by light scattering using a laser scattering microscope with a video camera. A video was taken, and the NTA software tracked the Brownian motion of the individual vesicles, calculating their size and concentration. Analysis of EVs by NTA revealed EVs that were approximately the same size in diameter at the 24-h and 48-h time points (Figure 2A,B). NTA revealed that at 24 h of EtOH administration, the EV particle number was unchanged (Figure 2C). NTA revealed that at 48 h of EtOH administration the EV particle number was slightly increased (Figure 2D). A representative histogram plot of control exosomes collected at 48 h indicated a mean size of 105.0 nm ± 7.9 nm and a particle count of 5.94 × 10^7^ particles/mL (Figure 2E).

To further confirm successful EV isolation and purification, enzyme-linked immunosorbent assay (ELISA) and SDS-PAGE/Western blot analyses were performed. The proteins of interest are well-known EV-associated proteins, CD81, Hsp70, and clathrin. Equal amounts of EVs from the 48 h samples (control, 50 mM, and 100 mM EtOH) were evaluated. The data demonstrated that the exosomal marker, CD81, was found in all HeLa-derived EVs. EVs derived from HeLa cells after 48 h of treatment with 100 mM EtOH significantly expressed CD81 ** *p* ≤ 0.01 (Figure 2F). The data also illustrated that Hsp70 (70 kDa) and clathrin (180 kDa) proteins were present in all EV samples (Figure 2G1,2). These data clearly represented the isolation of HeLa-derived EVs.

### 3.3. Expression of Rab Proteins

**Figure 3:**
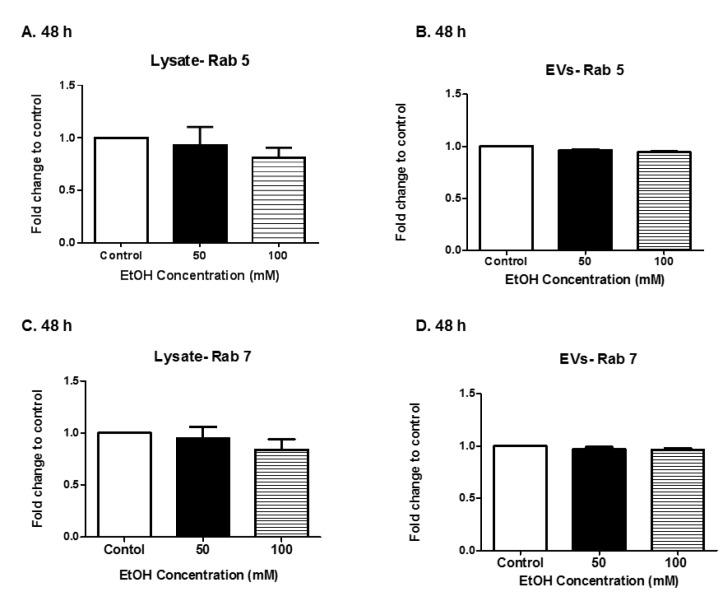
Expression of Rabs. (**A**,**C**) Cell lysate and (**B**,**D**) EV-associated proteins were evaluated at 48 h of EtOH administration for Rab 5 and Rab 7. In order to obtain quantitative results, cell lysates were subjected to dot blot analysis, and EV-associated proteins were subjected to ELISA. Data are presented as means ± SEM of five independent experiments.

Rabs are a group of GTPase proteins that are involved in a variety of biogenesis-related functions including membrane trafficking, vesicle formation, and secretion. We evaluated Rab 5 and Rab 7 expression in HeLa cell lysates and HeLa derived-EVs after EtOH administration. Cell lysates at the 48 h time point were selected for evaluation. A slight decrease in Rab 5 and Rab 7 protein expression in HeLa cell lysates at 48 h of 100 mM EtOH treatment was observed (Figure 3A,C). Equal amounts of Rab proteins were found within the EVs at 48 h post-EtOH treatment (Figure 3B,D). It is well documented that Rab 5 and Rab 7 proteins are found in various types of EVs [42]. These data clearly demonstrate the presence of EV-associated proteins.

### 3.4. Alcohol Dosing Increases Heat Shock Proteins within EVs

**Figure 4:**
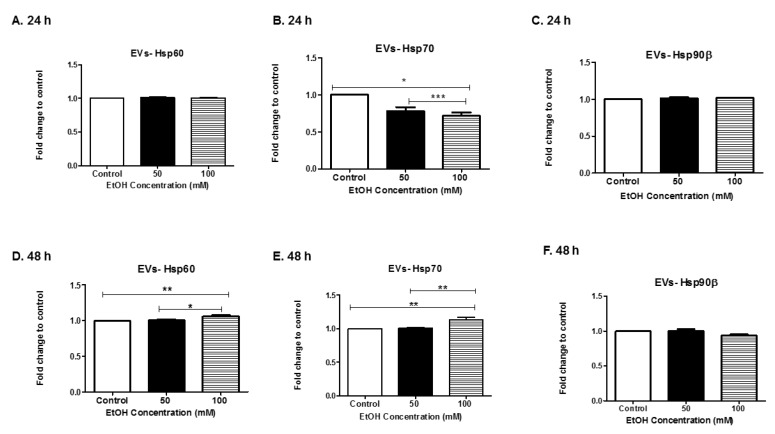
Production of heat shock proteins into HeLa-derived EVs. (**A**,**D**) Hsp60, (**B**,**E**) Hsp70, and (**C**,**F**) Hsp90β were observed in EVs after EtOH dosing at 24 h or 48 h using ELISA. Data are presented as the means ± SEM of eight independent experiments. Significance is defined as * *p* ≤ 0.05, ** *p* ≤ 0.01, and *** *p* ≤ 0.001.

Hsps are molecular chaperone proteins that mediate the synthesis and folding of proteins. They are induced in response to environmental stimuli and stressors such as alcohol [43,44]. In addition, cancer cells also secrete EVs that carry Hsps, which can alter tumor progression [45,46,47]. Therefore, we evaluated the levels of HeLa-derived EVs expressing Hsps60, 70, and 90 beta (β) (Figure 4A–F). We observed that Hsp60 in HeLa-derived EVs was detected at 24 h post-EtOH treatment (Figure 4A). At 48 h, Hsp60 levels within exosomes increased significantly in an EtOH dose-dependent manner (Figure 4D). Hsp70 was downregulated significantly in EVs derived after EtOH treatment at 24 h (Figure 4B) and significantly upregulated at 48 h of EtOH treatment (Figure 4E). Hsp90β was found in HeLa-derived EVs at either time point, EtOH exposure did not significantly impact Hsp90β expression in EVs (Figure 4C,F). Our results indicate that EtOH altered EV packaging of Hsps.

### 3.5. Alcohol Dosing Alters Apoptotic Proteins

**Figure 5:**
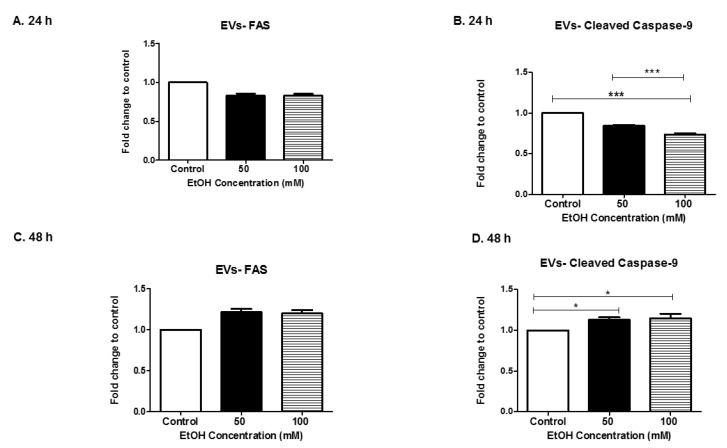
Expression of apoptotic proteins in EVs. FAS and cleaved caspase 9 were expressed in EVs after EtOH dosing at (**A**,**B**) 24 h and (**C**,**D**) 48 h and validated by means of ELISA. Data are presented as the means ± SEM of eight independent experiments. Significance is defined as * *p* ≤ 0.05 and *** *p* ≤ 0.001.

The *FAS* gene (full form) provides instructions for making a protein that is involved in cell signaling. Three FAS proteins form a trimer that then interacts with other molecules to perform its signaling function. This signaling starts a process known as the “caspase cascade”. The caspase cascade is a sequence of steps that results in the self-destruction of cells (apoptosis) when they are not needed [48,49]. At the early time point of 24 h, FAS expression in (50 mM and 100 mM) EtOH-derived EVs was decreased when compared to the control-derived EVs (Figure 5A). However, at the later time (48 h), FAS expression in (50 mM and 100 mM) EtOH-derived EVs increased over the control (Figure 5C). FAS proteins were modulated in EVs overtime in an EtOH dose-dependent manner (Figure 5A,C). Caspases are involved in regulating cell death in cells undergoing stress. Caspases are activated in a variety of conditions, (i.e., infections and chemical stimuli) [22,31,50]. The presence of cleaved caspase 9 indicate the active form of the protein, which causes an apoptotic cascade. Levels of cleaved caspase 9 in HeLa-derived EVs were significantly downregulated in EVs in an EtOH dose-dependent manner at 24 h (*p* ≤ 0.001, *p* ≤ 0.001) (Figure 5B). Levels of cleaved caspase 9 in HeLa-derived EVs were significantly upregulated in EVs in a time-dependent and EtOH dose-dependent manner at 48 h (*p* ≤ 0.05, *p* ≤ 0.05) (Figure 5D). These results were similar to the FAS results seen in Figure 5A,C. These results were consistent with the paradigm that FAS starts the caspase cascade. Our results indicate that alcohol modulates trafficking of apoptotic proteins within EVs.

## 4. Discussion

Despite a decrease in the prevalence of cervical cancer in developing countries, cervical cancer is still the second highest cancer among women [51]. In recent years, human papillomaviruses (HPV) has been used as a potential indicator of abnormal and precancerous cells in the cervical mucosa. HPV has over 120 different serotypes that can infect the skin and mucosa; 30 of those serotypes can infect the genital tract [52], and 13–15 of them are found in cancerous cervical cells. Globally, HPV 16 and HPV 18 are associated with approximately 70% of cervical cancer cases [53]. HPV is common in women in their early 20s and can go undetected due to the infection being self-limiting.

Alcohol consumption has been shown to increase the acquisition and pathogenesis of some viruses, such as human immunodeficiency virus [54,55,56,57]. The relationship between cervical cancer progression and alcohol consumption is a multifaceted process that is still being investigated. However, there is still a need for additional diagnostic tools to assess cancer status and stage. Once such tool/biomarker could be the use of EVs as a prognostic marker [4,10]. Therefore, in this study, we treated HeLa cells (HPV+) with 50 mM and 100 mM amounts of EtOH and evaluated various biological parameters. These concentrations of alcohol were chosen based on 100 mM being equivalent to one half of an average bottle of beer (1 M of beer is roughly equal to four average bottles of beer). Consumption of eight bottles of beer at 12-proof is comparable to the 200-proof EtOH we used in our study. In addition, in a previous study, human vein endothelial cells (HUVECs) treated with ethanol concentrations greater than 100 mM showed a significant change in cell toxicity [58].

Our results showed that EtOH administration significantly sensitized HeLa cells, and a noticeable reduction in HeLa viability at 50 mM and 100 mM concentrations at a time point of 48 h exposure when compared to control cells (Figure 1). EVs were isolated using standard methods and characterized after HeLa cells were dosed with EtOH [31,59]. EV quantities slightly increased at 48 h 100 mM of EtOH administration as compared to control (Figure 2D). Although we noticed the greatest cell death at a time point of 48 h EtOH exposure and a slight decreased in EV quantities, we observed an increase in various Hsps at this time point. We do acknowledge that there is a fair proportion of apoptotic bodies in our EV preparation since there was some cell death in our samples. However, these fractions should be mostly excluded from our sample populations due to filtration and differential ultracentrifugation. Our findings herein are similar to those by Momen-Heravi and group, who demonstrated that the hepatocyte cell line (Huh7.5) treated with EtOH (25 mM, 50 mM, and 100 mM) for 48 h yielded significant amounts of exosomes as compared to control cells. In addition, Momen-Heravi and group also demonstrated that primary hepatocytes treated with EtOH for 48 h yielded significant amounts of exosomes when compared to control cells [30]. These data are contrary to the data we observed for microglia BV-2-derived exosomes under the same conditions; EtOH treatment substantially decreased exosome production at 48 h [60]. Of important note, HeLa-derived EVs produced at 48-h administration of EtOH contained significant amounts of CD81. This is an important finding because tetraspanins’ expression within EVs can act as receptors, allowing EVs to enter cells. In addition, tetraspanins are also involved in cargo sorting of molecules within EVs [50,61]. We speculate that the abundance of CD81 will allow EVs to shuttle between neighboring cells delivering EVs cargo related to the physiological stressors of EtOH.

Rab 5 and Rab 7 are characteristically associated with the early endosome and late endosomes. The data illustrated a slight decrease in Rab 5 and Rab 7 protein expression in cell lysates at 48 h, 100 mM EtOH treatment (Figure 3A,C). This result was not surprising since exposure to a stimulus such as alcohol could cause an increase in vesicle recycling. However, at the 48-h time point, Rab 5 and Rab 7 protein expression remained unchanged in HeLa derived-EVs (Figure 3B,D). This finding is similar to what was observed in EtOH BV-2-derived exosomes [60]. Schulze et al. showed that EtOH exposure to rats decreased Rab 7 in hepatocytes [62], thus emphasizing the cell type results seen in this study herein.

Various Hsps were detected in HeLa-derived EVs, particularly Hsp60 (Figure 4A,D). We observed that Hsp60 in HeLa-derived EVs was detected at 24 h post-EtOH treatment. Furthermore, at 48 h, Hsp60 levels within EVs increased significantly in an EtOH dose-dependent manner. Our results were similar to Malik et al. This group studied cardiac myocyte-derived exosomes, specifically EtOH-derived exosomes. They found that EtOH did not affect the stability of cardiac myocyte-derived exosomes, but EtOH did greatly increase their production [63]. Additionally, their study found that Hsp60 is mainly linked with the exosomal membrane [64,65]. Interestingly, Hsp60 in EtOH-BV-2-derived was not observed [60], once again indicating that EtOH-exosome-dependent responses can be cell-type specific. As it relates to cancer/carcinogenesis, Hsps can be active players in carcinogenesis, as well as a promising target for anticancer therapy [36,66,67,68,69]. Studies by Campanella et al. postulated that Hsp60 released by tumor cells through exosomes could interact with peritumoral cells, as well as reach the bloodstream [70]. Additional studies by this research team found that among patients with colon cancer, Hsp60 is present in the pericellular interstitium of affected tissue, localizes on macrophages and NK cells, and concomitantly is found in the bloodstream of patients. Exosome analysis via body fluids might serve as a non-invasive predictor of cancer stage and success of cancer treatment.

Of the Hsps investigated in these studies, we observed the greatest range of expressions within Hsp70. Hsp70 protects brain cells against ischemia and other stressors [71]. One mechanism of Hsp70 protection is due to its ability to prevent damaging pro-inflammatory responses [72]. This may be the same phenomenon occurring with respect to HeLa cells. It is speculated that Hsp70 is upregulated at the later time point in an attempt to increase the likelihood of cell survival (Figure 4E). Similar results were seen by two independent groups. First, Wang and colleagues illustrated that the stress of electric stimulation induced distinct Hsp70 responses at both the mRNA and protein levels [73]. Second, Bharati et al.’s data suggested that the biphasic expression pattern of Hsp70 could be useful for helping animals in heat stress-induced situations, as well as serving as a biomarker of chronic heat stress in Tharparkar cattle [74]. Hsp70 is the most sensitive and considered as an important regulator of thermal adaptation during thermal stress of livestock [75].

Hsp90 has been found to be released in EVs by cancer cells [76,77]. Hsp90 can promote tumor growth and metastasis in breast cancer, pancreatic cancer, leukemia, and ovarian cancer [45,78,79]. Hsp90 is composed of a number of proteins including cytoplasmic Hsp90α, an inducible type, and Hsp90β, a constitutively-expressed type, as well as mitochondrial TRAP1. Although their expression levels increase under stressful conditions and in cancer cells, Hsp90β is one of the most abundant proteins in unstressed cells. This may account for why there was no change in Hsp90β expressing EVs derived after EtOH stimulus. Future investigation of the impact of EtOH on EVs’ expression of pan-cancer biomarkers Hsp90α and small Hsps (Hsp 16.2, 20 22, 27, alpha crystalline, and alpha-B crystalline) may yield useful information [45].

Both FAS and cleaved caspase 9 (Figure 5) are altered in EVs derived after EtOH treatment. Caspases have been shown to be important in EV-mediated cell-to-cell communication. Our results illustrate a strong biphasic expression of caspase 9 in EVs after EtOH exposure to HeLa cells. Along these same lines, Carboni et al. showed that the stress of ischemia induced biphasic caspase-9 activation in the hippocampus of Mongolian gerbils [80]. Studies by Vardaki et.al, demonstrate that cleavage of caspase 3, causes uptake of recipient cells [63]. Data presented by several studies and our group indicate that EtOH-mediated EVs responses are cell-type specific.

## 5. Conclusions

Our data illustrate that EtOH modulates cellular biogenesis of HeLa cells. Specifically, EtOH modulates EV biogenesis and the cargo of HeLa derived-EV. Although HeLa cells used the primary model in this study, the research findings may have substantial implications on diagnostics and therapy for a variety of cell types and organ systems. Further investigation is needed to elucidate the mechanism(s) involved in these processes.

## Data Accessibility

The original data will be maintained by the corresponding author. Information pertaining to the datasets will be made available upon written request.
